# Effect of Tauroursodeoxycholic Acid and 4-Phenylbutyric Acid on Metabolism of Copper and Zinc in Type 1 Diabetic Mice Model

**DOI:** 10.1007/s12011-015-0474-5

**Published:** 2015-08-19

**Authors:** Qi Zhou, Di Wang, Jiancheng Xu, Baorong Chi

**Affiliations:** Department of Pediatrics, First Hospital of Jilin University, Changchun, 130021 China; Department of Laboratory Medicine, First Hospital of Jilin University, 71 Xinmin Street, Changchun, 130021 China; Department of Hepatology, First Hospital of Jilin University, 71 Xinmin Street, Changchun, 130021 China

**Keywords:** Tauroursodeoxycholic acid, 4-phenylbutyric acid, Zinc, Copper, Diabetes

## Abstract

Alternations of copper (Cu) and zinc (Zn) status in diabetes have received a great attention. Tauroursodeoxycholic acid (TUDCA) and 4-phenylbutyric acid (PBA) could alleviate the increased endoplasmic reticulum (ER) stress and prevent insulin resistance. This study aimed to investigate the effect of TUDCA and PBA on metabolism of Cu and Zn in diabetic mice model. Diabetes was induced by streptozotocin in FVB mice treated with and without TUDCA and PBA. Determination of Cu and Zn in tissues and serum by acid digestion was followed by ICP-MS. The renal and serum Cu levels were significantly higher, while the hepatic Cu and Zn levels were significantly decreased in the diabetic mice at 2 weeks and 2 months after diabetes onset. The increase of cardiac Cu together with the decrease of muscular Zn was found in the diabetic mice only at 2 months. Cu levels were positively correlated with Zn in the heart, liver, kidney, muscle, spleen, and serum of diabetic and control mice at both 2 weeks and 2 months. Both PBA and TUDCA reduced serum Zn, and PBA reduced hepatic Cu to normal levels in the diabetic mice at two time points, while PBA normalized serum Cu in the diabetic mice only at 2 months. PBA increased hepatic Zn to normal levels in the diabetic mice at 2 weeks, while it partially increased hepatic Zn in the same group at 2 months. Therefore, maintaining homeostasis of Cu and Zn by TUDCA and PBA in diabetes needs to be received with special attention.

## Introduction

Diabetes mellitus (DM) has become one of the most severe endocrine metabolic disorders in the world. Diabetes damages multiple organs to induce serious complications such as diabetic retinopathy, neuropathy, and nephropathy that can result in the disability and mortality for diabetic patients [1]. Excessive caloric intake and high-energy diet quality are major driving forces behind escalating diabetes and the appearance of epidemics worldwide [2]. As an essential component of the daily diet intake, trace elements are important for the occurrence and progression of diabetes. Alternations in trace element status and increased oxidative stress in diabetes may contribute to insulin resistance and the development of diabetes and diabetic complications [3, 4]. On the other hand, progression of diabetes may also lead to perturbation in trace element metabolism and homeostasis [5].

Cu and Zn are prooxidants, playing critical roles in metal-catalyzed formation of free radicals. In the presence of copper ions, a hydroxyl radical, which is the most powerful reactive oxygen species (ROS), can be produced via the Fenton or the metal-catalyzed Haber-Weiss reaction [6]. These two chemical reactions appear to account for most of the hydroxyl radical production in biological systems and explain, at least in part, why Cu produces oxidative stress and ROS-induced injury in cells. Cu and Zn also act as structural and catalytic components of some metalloenzymes [5]. For example, Cu is necessary for the catalytic activity of Cu/Zn superoxide dismutase (SOD) [5], extracellular superoxide dismutase (EC-SOD) [7], cytochrome c oxidase (COX) [8], and ceruloplasmin/ferroxidase [9]. Zn acts as an antioxidant by protecting the sulfhydryl groups of proteins and enzymes against free radical damage in the body [10]. Abnormal Cu metabolism is associated with human and experimental diabetes. Cu deficiency can cause neurodegeneration [11] and hematological and cardiovascular disorders [12], whereas Cu overload may be accompanied by hepatic and neurological diseases [13, 14]. Recently, we found the serum Cu level was significantly higher in the patients with impaired fasting glucose, impaired glucose tolerance, and type 2 diabetes, compared to control subjects [15]. The contribution of alternations in the homeostasis ions of these metals on the occurrence and progression of diabetes is still being discussed. The significance of variable concentrations of these trace metals in different organs of streptozotocin (STZ)-induced diabetic mice is discussed.

Chemical or pharmaceutical chaperones, such as tauroursodeoxycholic acid (TUDCA) and 4-phenylbutyric acid (PBA), could alleviate the increased endoplasmic reticulum (ER) stress [16]. Recently, several papers focus on the relationship between the chaperones and calcium. A study indicates both PBA and TUDCA inhibit the reduction in mitochondrial calcium and decrease in mitochondrial reactive oxygen species (mROS) despite ongoing hypoxia in pulmonary artery smooth muscle cells of rats [16]. TUDCA significantly increases hepatic calcium content and serum calcium concentration after ischemia-reperfusion in rat [17]. TUDCA markedly prevents aortic valve calcification in both rabbit and mouse models of aortic valve calcification [18]. There was no much information for the effect of chaperones on metabolism of Cu and Zn in diabetic mice model.

This study aimed to investigate the effect of TUDCA and PBA on metabolism of Cu and Zn in diabetic mice model. We supposed that TUDCA and PBA could be effective in the maintenance of homeostasis of Cu and Zn in diabetes. In the present study, we measured the changes of Cu and Zn concentrations in the heart, liver, kidney, muscle, spleen, and serum in type 1 diabetic mice treated with and without chaperones.

## Materials and Methods

### Animal Models

Male Friend virus B (FVB) mice, 8 weeks of age, purchased from Vital River Laboratories (Beijing, China), were housed in the Experimental Animal Center of College of Basic Medical Sciences of Jilin University at 22 °C with a 12-h light/dark cycle and free access to standard rodent chow and tap water. All animal procedures were approved by the Institutional Ethics Committee of the First Hospital of Jilin University. The mice were randomly divided into six groups (*n* = 14 in each group), namely, nondiabetic control mice (CON), diabetic mice (DM), diabetic mice treated with PBA (PBA + DM) or TUDCA (TUDCA + DM), and nondiabetic control mice treated with PBA (PBA) or TUDCA (TUDCA). Type 1 diabetic mice were induced by intraperitoneal injection of multiple low-dose STZ (40 mg/kg body weight daily for 5 days; Sigma Chemical Co., St. Louis, MO, USA) dissolved in a sodium citrate buffer (pH 4.5). Five days after the last injection of STZ, whole blood obtained from each mouse’s tail vein was used for glucose monitoring with the use of a glucometer (Bayer HealthCare, Mishawaka, IN, USA). STZ-treated mice with whole-blood glucose levels higher than 12 mmol/L were considered diabetic. Two days before the last injection of STZ, acclimation was started with daily phosphate-buffered saline (PBS) administration. The groups used for PBA (Merck KGaA, Hohenbrunn, Germany) treatments served 100 μL PBS twice per day (8 am and 8 pm) by gavage and the groups used for TUDCA (Calbiochem, La Jolla, CA, USA) treatments received intraperitoneal injection of 100 μl PBS at the same time points (8 am–8 pm) for 3 days. Three days later, at 8 am, blood samples were collected from the tail vein for measurement of glucose levels and treatments were initiated with the vehicle or the drugs. This time point was taken as day zero. PBA was administered two times a day in two divided doses (500 mg/kg for 8 am and 8 pm, total 1 g/kg/day) by oral gavage. TUDCA was applied intraperitoneally at the same time points (250 mg/kg for 8 am and 8 pm, total 500 mg/kg/day). Controls for PBA, received the same volume of vehicle by gavage and controls for TUDCA-treated group received the same volume of vehicle by intraperitoneal injection. Body weight and blood glucose levels were measured regularly.

Animals from each group were sacrificed 2 weeks and 2 months after the administration of PBA or TUDCA (*n* = 7 in each time point of each group). The animals were anesthetized with 2 % sodium pentobarbital (30 mg/kg, intraperitoneal) and were sacrificed by cardiac puncture. Heart, liver, kidney, spleen, and soleus muscles were harvested and stored at −80 °C. Blood samples through the retro-orbital plexus were taken into special metal-free tubes for analysis of laboratory parameters. After blood centrifugation, serum was aliquoted into metal-free Eppendorf test tubes, shock-frozen, and stored at −80 °C for later analysis.

### Determination of Cu and Zn by Acid Digestion Followed by ICP-MS

Preweighed pieces of tissue samples (approximately 0.5–1 g of tissue) from the above experiments were digested separately in 1 mL nitric acid in a 10-mL glass vial at room temperature and then heated to 110 °C for 8 h to facilitate digestion. A volume of 4 mL deionized water was added to each vial to increase the volume to 5 mL. The resulting clear liquid was used for analysis. Cu and Zn concentrations were determined by Agilent Technologies 7700 Series ICP-MS equipment (Agilent Technologies, Santa Clara, CA, USA). The instrument was tuned on a daily basis to ensure optimisation. Rf Power was 1550 W and nebulizer gas flow rate was kept at 1.05 L/min. A heating program appropriated to each type of sample was applied. Cu and Zn concentrations were expressed as micrograms per liter wet tissue.

### Other Measurements

Laboratory indices including glycosylated serum protein (GSP), blood urea nitrogen (BUN), creatinine (Cre), uric acid (UA), total cholesterol (CHO), triglyceride (TG), high-density lipoprotein (HDL), and low-density lipoprotein cholesterol (LDL) levels were performed by Hitachi 7600-010 Clinical Chemistry Analyzer (Hitachi, Tokyo, Japan) using standard methodology from the blood samples, which were obtained at the time of sacrifice.

### Statistical Analysis

Continuous variables were expressed as mean ± SE. Student’s *t* test was used for comparisons between groups. Spearman rank correlation analysis was used to evaluate the correlations between serum Cu and Zn level as a continuous variable and laboratory parameters. All reported *p* values were two-sided, and values of *p* < 0.05 were considered statistically significant. Statistical analyses were performed using SPSS 17.0.

## Results

### Baseline Characteristics

Baseline characteristics of type 1 diabetic mice were summarized in Table 1. Decreased body weight and increased blood glucose were seen in the diabetic mice at both 2 weeks and 2 months after the onset of diabetes. However, TUDCA or PBA inhibited the trends of body weight and blood glucose in the diabetic mice, although the body weight and blood glucose could not restore normal level. Both GSP and BUN levels were significantly increased in the diabetic mice at 2 months and TUDCA or PBA did not show any effect on them. Cre was significantly increased in the diabetic mice at 2 months, but not diabetic mice treated with PBA. TUDCA reduced CHO and LDL to normal levels in the diabetic mice at 2 months. TUDCA and PBA partially suppressed the increase of TG and LDL in the diabetic mice at 2 months, respectively. Compared to control, the serum of Cu level was significantly higher in the diabetic mice at both 2 weeks and 2 months after the onset of diabetes. PBA reduced Cu to normal levels in the diabetic mice at 2 months. There were no significant differences among all groups for the following serum laboratory parameters: UA and HDL.

**Table 1:** Baseline characteristics in type 1 diabetic mice

	2 weeks						2 months					
	CON	PBA	TUDCA	PBA + DM	TUDCA + DM	DM	CON	PBA	TUDCA	PBA + DM	TUDCA + DM	DM
BW (g)	28.7 ± 0.23	29.1 ± 0.46	28.9 ± 0.30	28.8 ± 0.49^※^	26.0 ± 0.35^§※^	23.6 ± 0.78*	33.4 ± 0.62	33.6 ± 0.33	33.7 ± 0.53	25.8 ± 0.28^#※^	26.1 ± 0.20^§※^	23.1 ± 0.25*
GLU (mmol/L)	7.2±0.45	7.2 ± 0.42	7.2 ± 0.39	26.4 ± 0.47^#※^	21.4 ± 0.84^§※^	31.9 ± 0.64*	8.8 ± 0.24	8.9 ± 0.13	8.6 ± 0.21	28.5 ± 0.76^#※^	23.1 ± 0.71^§※^	33.7 ± 0.61*
GSP (μmol/L)	−	−	−	−	−	−	259.6 ± 17.9	260.3 ± 10.2	261.1 ± 9.4	460.6 ± 11.5^#^	431.7 ± 11.9^§^	475.4 ± 19.2*
BUN (mmol/L)	−	−	−	−	−	−	11.3 ± 0.39	11.3 ± 0.32	11.5 ± 0.40	16.1 ± 0.31^#^	15.3 ± 0.55^§^	16.8 ± 0.59*
Cre (mmol/L)	−	−	−	−	−	−	12.7 ± 0.28	12.3 ± 0.28	12.2 ± 0.24	13.3 ± 0.94^※^	18.4 ± 0.84^§^	20.5 ± 2.30*
UA (μmol/L)	−	−	−	−	−	−	133.9 ± 2.1	133.6 ± 2.2	134.3 ± 2.5	130.0 ± 5.2	140.9 ± 3.4	134.3 ± 8.6
H-Cu (mg/L)	7.0 ± 0.19	6.9 ± 0.16	6.9 ± 0.16	7.2 ± 0.26	7.3 ± 0.34	6.6 ± 0.23	7.2 ± 0.24	7.2 ± 0.24	7.2 ± 0.23	6.3 ± 0.09*	6.5 ± 0.16^§^	6.4 ± 0.22*
H-Zn (mg/L)	17.6 ± 0.66	17.4 ± 0.71	17.3 ± 0.69	19.9 ± 1.23	18.4 ± 0.75	19.1 ± 0.67	18.3 ± 0.48	18.3 ± 0.66	18.2 ± 0.58	18.7 ± 1.37	18.7 ± 0.78	18.6 ± 0.68
L-Cu (mg/L)	7.7 ± 0.27	7.6 ± 0.22	7.7 ± 0.28	7.4 ± 0.13^※^	6.8 ± 0.15^§^	6.5 ± 0.15*	7.5 ± 0.28	7.5 ± 0.26	7.5 ± 0.24	7.5 ± 0.31^※^	6.4 ± 0.18^§^	6.3 ± 0.44*
L-Zn (mg/L)	52.1 ± 3.01	52.7 ± 2.05	53.3 ± 2.23	51.7 ± 2.10^※^	42.6 ± 0.64^§^	41.0 ± 1.32*	56.7 ± 2.02	54.2 ± 2.17	56.0 ± 2.08	42.7 ± 1.69^#※^	34.3 ± 0.91^§^	33.9 ± 1.76*
K-Cu (mg/L)	5.6 ± 0.16	5.7 ± 0.17	5.5 ± 0.13	6.6 ± 0.16^#^	6.0 ± 0.07^§^	6.4 ± 0.20*	5.3 ± 0.18	5.3 ± 0.17	5.3 ± 0.16	7.1 ± 0.20^#^	6.7 ± 0.25^§^	7.4 ± 0.66*
K-Zn (mg/L)	21.7 ± 0.66	21.7 ± 0.75	21.6 ± 0.61	20.7 ± 0.82	22.2 ± 0.36	20.5 ± 0.53	18.8 ± 0.22	19.4 ± 0.53	18.9 ± 0.40	20.5 ± 1.22	19.1 ± 0.99	21.1 ± 2.02
M-Cu (mg/L)	1.17 ± 0.03	1.14 ± 0.04	1.16 ± 0.04	1.14 ± 0.03	1.11 ± 0.04	1.11 ± 0.03	1.11 ± 0.03	1.11 ± 0.04	1.09 ± 0.04	1.00 ± 0.03	1.09 ± 0.04	1.10 ± 0.04
M-Zn (mg/L)	10.5 ± 0.30	10.5 ± 0.27	10.2 ± 0.27	11.2 ± 0.37	11.1 ± 0.32	11.3 ± 0.3	10.1 ± 0.34	10.1 ± 0.34	9.9 ± 0.31	14.2 ± 0.42^#^	14.1 ± 0.44^§^	15.3 ± 1.07*
P-Cu (mg/L)	1.29 ± 0.04	1.33 ± 0.08	1.34 ± 0.08	1.30 ± 0.09	1.36 ± 0.06	1.36 ± 0.19	1.27 ± 0.03	1.29 ± 0.03	1.28 ± 0.02	1.09 ± 0.06	1.23 ± 0.05	1.27 ± 0.09
P-Zn (mg/L)	26.7 ± 0.50	26.2 ± 0.43	27.0 ± 1.12	26.4 ± 1.35	27.6 ± 0.92	27.7 ± 0.38	27.3 ± 0.56	27.4 ± 0.50	26.2 ± 0.73	24.9 ± 0.59	25.9 ± 0.93	27.1 ± 1.53
S-Cu (mg/L)	1.08 ± 0.74	1.09 ± 0.76	1.10 ± 0.18	1.34 ± 0.12^#^	1.37 ± 0.37^§^	1.39 ± 0.70*	1.08 ± 0.62	1.09 ± 0.60	1.08 ± 0.52	1.04 ± 0.31^※^	1.37 ± 0.34^§^	1.42 ± 0.12*
S-Zn (mg/L)	1.48 ± 0.45	1.48 ± 0.43	1.48 ± 0.43	1.43 ± 0.41	1.38 ± 0.30	1.37 ± 0.31	1.40 ± 0.45	1.41 ± 0.44	1.40 ± 0.48	1.43 ± 0.41^※^	1.42 ± 0.32^※^	1.26 ± 0.60
CHO (mmol/L)	−	−	−	−	−	−	3.9 ± 0.16	3.9 ± 0.14	3.9 ± 0.12	5.9 ± 0.62^#^	4.1 ± 0.19^※^	6.9 ± 0.60*
TG (mmol/L)	−	−	−	−	−	−	3.3 ± 0.37	3.2 ± 0.12	3.0 ± 0.13	6.1 ± 0.61^#^	4.6 ± 0.21^§※^	6.9 ± 0.74*
HDL (mmol/L)	−	−	−	−	−	−	2.8 ± 0.07	2.8 ± 0.07	2.9 ± 0.07	3.4 ± 0.19	2.9 ± 0.10	3.4 ± 0.24
LDL (mmol/L)	−	−	−	−	−	−	0.81 ± 0.05	0.81 ± 0.06	0.81 ± 0.03	1.39 ± 0.15^#※^	0.76 ± 0.03^※^	2.04 ± 0.25*

Data are presented as mean ± SE, *n* = 7 in all groups

*BW* body weight, *Glu* serum glucose, *GSP* glycosylated serum protein, *BUN* blood urea nitrogen, *Cre* serum creatinine, *UA* uric acid, *H-Cu* copper concentration in heart, *H-Zn* zinc concentration in heart, *L-Cu* copper concentration in liver, *L-Zn* zinc concentration in liver, *K-Cu* copper concentration in kidney, *K-Zn* zinc concentration in kidney, *M-Cu* copper concentration in muscle, *M-Zn* zinc concentration in muscle, *P-Cu* copper concentration in spleen, *P-Zn* zinc concentration in spleen, *S-Cu* copper concentration in serum, *S-Zn* zinc concentration in serum, *CHO* total cholesterol, *TG* triglyceride, *HDL* high-density lipoprotein cholesterol, *LDL* low-density lipoprotein

*p < 0.05 vs CON group; ^§^ *p <* 0.05 vs TUDCA group; ^#^ *p <* 0.05 vs PBA group; ^※^ *p <* 0.05 vs DM group

### Analysis of Cu and Zn in the Heart, Liver, Kidney, Muscle, Spleen, and Serum of Type 1 Diabetic Mice Treated With and Without Chaperones

**Fig. 1:**
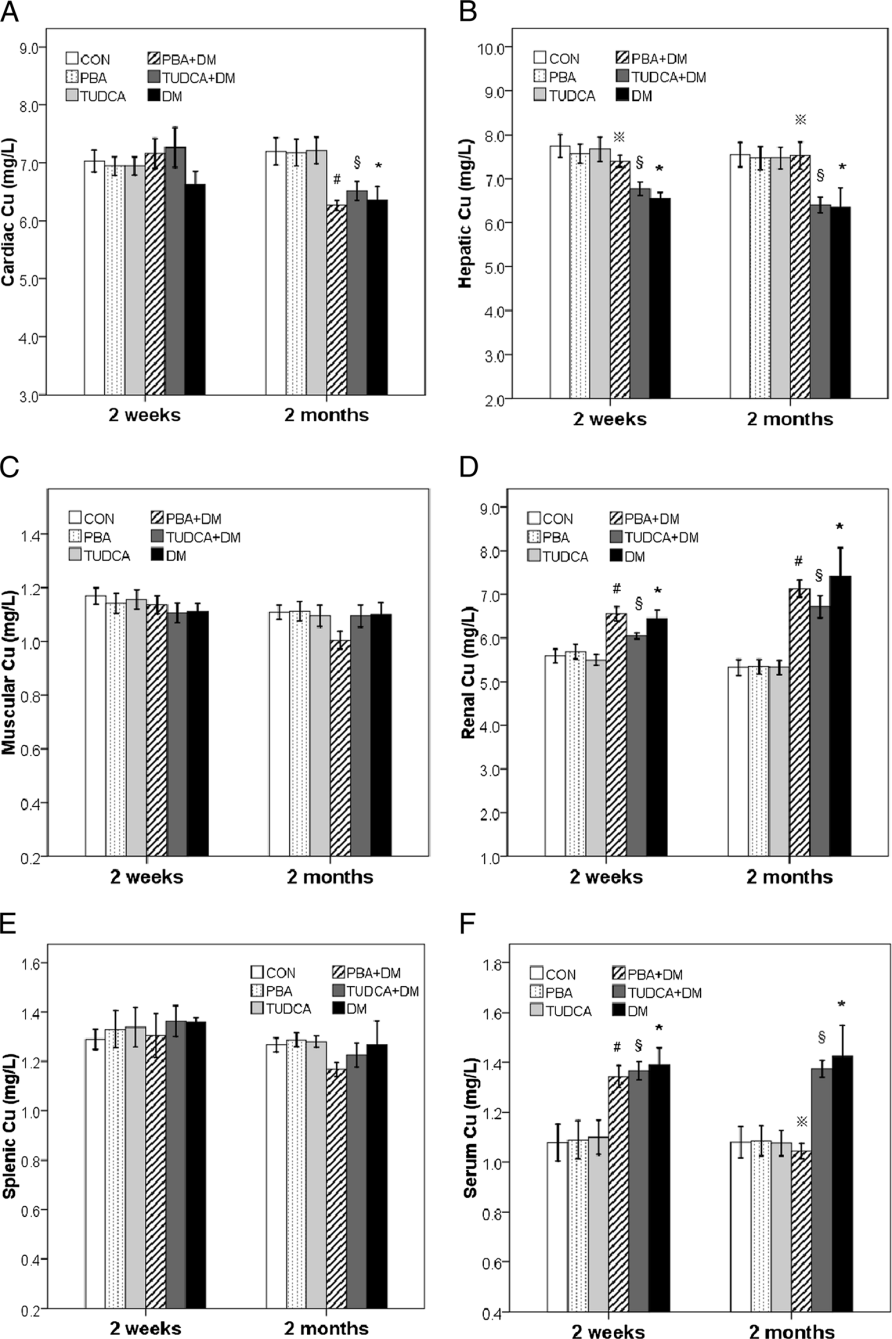
Levels of Cu in the heart, liver, kidney, muscle, spleen, and serum of type 1 diabetic mice model. Levels of Cu in the heart (**a**), liver (**b**), kidney (**c**), muscle (**d**), spleen (**e**), and serum (**f**) in 2 weeks and 2 months after the administration of PBA or TUDCA. Data are presented as mean ± SE, *n* = 7 in all groups. *CON* nondiabetic control group, *DM* diabetic mice group, *PBA* + *DM* group of diabetic mice treated with PBA, *TUDCA* + *DM* group of diabetic mice treated with TUDCA, *PBA* group of nondiabetic control mice treated with PBA, *TUDCA* group of nondiabetic control mice treated with TUDCA

Compared to respective control, the renal Cu level was significantly higher in the diabetic mice treated with and without chaperones both at 2 weeks and 2 months, while the cardiac Cu level was significantly lower and muscular Zn was significantly higher in the same groups only at 2 months. PBA increased hepatic Cu to normal levels in the diabetic mice both at 2 weeks and 2 months, while both PBA and TUDCA increased serum Zn, and PBA normalized serum Cu in the diabetic mice only at 2 months. PBA increased hepatic Zn to normal levels in the diabetic mice at 2 weeks, while it partially increased hepatic Zn in the same group at 2 months. There were no significant differences of Cu levels in the muscle and spleen, Zn levels in the heart, kidney, and spleen among all groups. The levels of Cu and Zn in the heart, liver, kidney, muscle, spleen, and serum of type 1 diabetic mice model are presented in Table 1 and Fig. 1.

### Associations Between Cu and Zn Level in the Heart, Liver, Kidney, Muscle, Spleen, and Serum of Type 1 Diabetic Mice Treated With and Without Chaperones

**Fig. 2:**
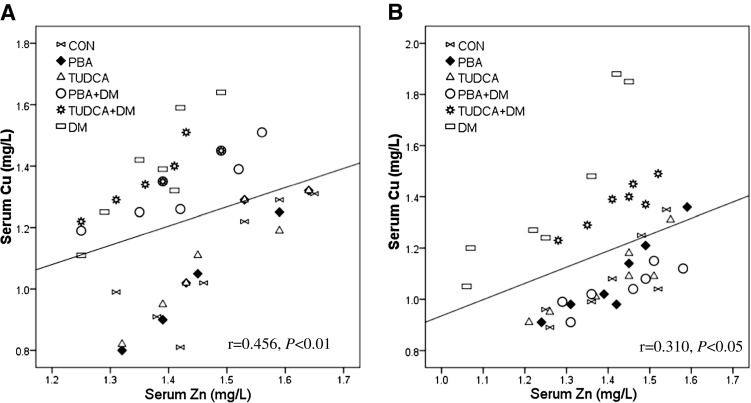
Correlation between serum Cu and serum Zn in type 1 diabetic mice model. Correlations between serum Cu and serum Zn in 2 weeks (*r* = 0.456, *p* < 0.01, **a**) and 2 months (*r* = 0.310, *p* < 0.05, **b**) after the administration of PBA or TUDCA

We analyzed associations between Cu and Zn in tissues and the serum of all groups (Tables 2 and 3 and Fig. 2). Overall, Cu levels were positively correlated with zinc in the heart, liver, kidney, muscle, spleen, and serum of all groups both at 2 weeks and 2 months except the spleen of diabetic mice treated with TUDCA at 2 weeks. The correlation curve between serum Cu and serum Zn in all groups is presented in Fig. 2.

**Table 2:** Associations between Cu and Zn level in the heart, liver, kidney, muscle, spleen, and serum of 2-week mice

	Cu											
	CON		PBA		TUDCA		PBA + DM		TUDCA + DM		DM	
	*r*	***p***	*r*	***p***	*r*	***p***	*r*	***p***	*r*	***p***	*r*	***p***
H-Zn	0.865	0.012*	0.955	0.001*	0.821	0.023*	0.857	0.014*	0.847	0.016*	0.857	0.014*
L-Zn	0.847	0.016*	0.893	0.007*	0.857	0.014*	0.811	0.027*	0.919	0.003*	0.893	0.007*
K-Zn	0.821	0.023*	0.811	0.027*	0.857	0.014*	0.901	0.006*	0.847	0.016*	0.857	0.014*
M-Zn	0.901	0.006*	0.786	0.036*	0.893	0.007*	0.964	<0.001*	0.847	0.016*	0.893	0.007*
P-Zn	0.821	0.023*	0.964	<0.001*	0.821	0.023*	0.955	0.001*	0.464	0.294	0.937	0.002*
S-Zn	0.857	0.014*	0.964	<0.001*	0.964	<0.001*	0.929	0.003*	0.964	<0.001*	0.857	0.014*

Abbreviations’ spellings are same as the description for Table 1

*p < 0.05 for the association

**Table 3:** Associations between Cu and Zn level in the heart, liver, kidney, muscle, spleen, and serum of 2-month mice

	Cu											
	CON		PBA		TUDCA		PBA + DM		TUDCA + DM		DM	
	*r*	*p*	*r*	*p*	*r*	*p*	*r*	*p*	*r*	*p*	*r*	*p*
H-Zn	0.893	0.007*	0.893	0.007*	0.821	0.023*	0.929	0.003*	0.893	0.007*	0.893	0.007*
L-Zn	0.857	0.014*	0.929	0.003*	0.929	0.003*	0.893	0.007*	0.821	0.023*	0.857	0.014*
K-Zn	0.937	0.002*	0.821	0.023*	0.821	0.023*	0.929	0.003*	0.964	<0.001*	0.544	<0.001*
M-Zn	0.786	0.036*	0.893	0.007*	0.821	0.023*	0.857	0.014*	0.893	0.007*	0.857	0.014*
P-Zn	0.847	0.016*	0.893	0.007*	0.883	0.008*	0.964	<0.001*	0.955	0.001*	0.929	0.003*
S-Zn	0.857	0.014*	0.937	0.002*	0.918	0.004*	0.929	0.003*	0.786	0.036*	0.929	0.003*

Abbreviations’ spellings are same as the description for Table 1

*p < 0.05 for the association

## Discussion

In the present study, decreased body weight and increased blood glucose were seen in the diabetic mice at both 2 weeks and 2 months after the onset of diabetes. These results are in accordance with the results previously reported after STZ treatment of diabetic mice [19]. The levels of GSP, BUN, Cre, CHO, TG, and LDL were significantly increased in the diabetic mice at 2 months, consistent with the previous findings [20, 21].

### Cu and Zn in Tissues and Serum

Diabetes results in several metabolic changes, including alterations in the transport, distribution, excretion, and accumulation of metals [22]. To our knowledge, this is the first study to systematically analyze Cu and Zn in the heart, liver, kidney, muscle, spleen, and serum of type 1 diabetic mice. In the present study, the renal and serum Cu levels were significantly higher, while the hepatic Cu and Zn levels were significantly decreased in the diabetic mice at 2 weeks and 2 months. The decrease of cardiac Cu together with the increase of muscular Zn was found in the diabetic mice only at 2 months. There were no significant differences of Cu levels in the muscle and spleen, Zn levels in the heart, kidney, spleen, and serum among all groups. The present study indicated an imbalance in amounts of Cu and Zn in STZ-diabetic mice compared to their levels in healthy subjects.

ROS are known to preferably attack lipids and lead to abnormalities in lipid metabolism in diabetes, while diabetic dyslipidemia induces oxidative load [23]. Free radical production is considered as one of the major mechanisms responsible for the toxicity of Cu [24]. Excessive tissue accumulation of redox-active Cu ions can be cytotoxic, in particular because perturbations in metal homeostasis result in an array of cellular disturbances characterized by oxidative stress and increased free radical production [6]. The increased serum and renal Cu may increase oxidative stress and subsequent inflammation, leading to the insulin resistance and development of diabetes. These findings may contribute to explain the role of impaired ion metabolism of Cu and Zn in the progression of diabetic oxidative complications. In the present study, the amounts of Cu and Zn were not consistent in tissues and serum at different time points. The blood usually represents a small, but rapidly mobilizable pool of the metal ion. In contrast, tissues, such as the liver, represent a large storage pool of the metal, which exchange only very slowly with the pool of metal in the bloodstream [22]. Our results imply perturbations in homeostasis of Cu and Zn in tissues and serum of STZ-induced diabetic mice may be involved in disturbances of oxidative balance. Therefore, these findings may contribute to explain the role of impaired ion metabolism of some elements in the progression of diabetic oxidative complications.

### Associations Between Cu and Zn Level in Tissues and Serum

We analyzed associations between Cu and Zn in tissues and serum of all groups. Interestingly, Cu levels were positively correlated with Zn in the heart, liver, kidney, muscle, spleen, and serum of all groups both at two time points except the spleen of diabetic mice treated with TUDCA at 2 weeks. In our previous study, serum Cu levels were positively correlated with serum Zn in control subjects and the patients with type 1 diabetes [15]. These results indicated that Cu levels were positively associated with Zn in tissues and serum in health and diabetes of human and mice model, which implied Cu and Zn may have regulatory effects on each other. Further clinical and animal investigations with larger numbers of cases are needed.

### Effect of TUDCA and PBA on Metabolism of Copper and Zinc

TUDCA is a bile acid existed in human bile at low concentration and has a very good safety profile. It has been used for centuries in traditional Chinese medicine (isolated from the dried bile of adult black bears) in the treatment of a variety of ailments. TUDCA also has been used widely in clinical applications in the Western World for treatment of biliary and liver diseases [25]. PBA has been approved by the U.S. Food and Drug Administration for clinical use in urea-cycle disorders as an ammonia scavenger and has been in clinical trials for the treatment of other diseases such as thalassemia and cystic fibrosis [26]. Both TUDCA and PBA have been attributed to antiapoptotic, anti-inflammatory, antioxidative, and immunomodulation effects [18, 25, 27]. A few studies indicate that TUDCA and PBA enhance the adaptive capacity of ER and prevent insulin resistance [25, 26].

In the present study, there was a small but statistically significant increase in body weight and a decrease in blood glucose in the TUDCA- or PBA-treated groups. When administered to diabetic mice, TUDCA and PBA exhibited a potent antidiabetic activity [26]. Recently, several papers focus on the relationship between the chaperones and calcium [16–18]. However, there has been no much information for the effect of chaperones on metabolism of copper and zinc in diabetic mice model. In the present study, both PBA and TUDCA increased serum Zn, and PBA increased hepatic Cu to normal levels in the diabetic mice at two time points, while PBA normalized serum Cu in the diabetic mice only at 2 months. PBA increased hepatic Zn to normal levels in the diabetic mice at 2 weeks, while it partially increased hepatic Zn in the same group at 2 months. Our results confirmed the chaperones extended a protective action in STZ-induced diabetes. To our knowledge, this is the first report to study the effects of TUDCA and PBA on the mineral distribution of tissues in type 1 diabetic mice.

There was no much information for the mechanism of chaperones on metabolism of copper and zinc in diabetic mice model. Recently, a study indicates both PBA and TUDCA inhibit the reduction in mitochondrial calcium and decrease in mROS [16]. We hypothesize that chaperones maintain homeostasis of Cu and Zn at least via their antioxidative effects. The detailed mechanism of chaperones maintaining homeostasis of trace elements in diabetes remains unclear and in need of further exploration.

There are several limitations of this work. First, because of the little number of mice in each group, there still remains uncertainty as to the results in this research. Second, because of not detecting laboratory indices of ROS, we could not evaluate the association between ROS and trace elements in tissues and serum of type 1 diabetic mice. Third, we did not investigate the mechanism of chaperones on metabolism of copper and zinc in diabetic mice model in detail. A well-designed study to address these limitations may be necessary in the future to indicate the effect of chaperones on metabolism of copper and zinc in clinical and animal investigations with larger numbers of cases.

## Conclusions

Alternations of Cu and Zn status in diabetes have received great attention. TUDCA and PBA could alleviate the increased ER stress and prevent insulin resistance. This is the first study to systematically analyze the effects of TUDCA and PBA on Cu and Zn distributions in tissues and serum in STZ-induced diabetic mice. We found the following: (1) Cu and Zn levels were imbalanced in the heart, liver, kidney, muscle, and serum of STZ-diabetic mice compared to their levels in healthy subjects; (2) Cu levels were positively correlated with Zn in the heart, liver, kidney, muscle, spleen, and serum of diabetic and control mice at both 2 weeks and 2 months after the onset of diabetes; (3) both PBA and TUDCA increased serum Zn, and PBA increased hepatic Cu to normal levels in the diabetic mice at two time points, while PBA normalized serum Cu in the diabetic mice only at 2 months. PBA increased hepatic Zn to normal levels in the diabetic mice at 2 weeks, while it partially increased hepatic Zn in the same group at 2 months. Therefore, maintaining homeostasis of Cu and Zn by TUDCA and PBA in diabetes needs to be received with special attention. We suggest that chemical chaperones in general, and PBA and TUDCA in particular, may warrant clinical investigation as regulators for imbalance of Cu and Zn in diabetes.
